# Brain-mechanistic responses to varying difficulty levels of approximate solutions to arithmetic problems

**DOI:** 10.1038/srep24194

**Published:** 2016-04-13

**Authors:** Yanhui Xiang, Yiqi Jiang, Xiaomei Chao, Qihan Wu, Lei Mo

**Affiliations:** 1 Guangdong Key Laboratory of Mental Health and Cognitive Science, South China Normal University, Guangzhou 510631, P. R. China; 2 School of Psychology, South China Normal University, Guangzhou, Guangdong, Guangzhou 510631, P. R. China; 3 Center for Studies of Psychological Application, South China Normal University, Guangzhou, Guangdong, 510631, P. R. China; 4 Xinhu School, Bao’an, Shenzhen, Guangdong, 518100, P. R. China

## Abstract

Approximate strategies are crucial in daily human life. The studies on the “difficulty effect” seen in approximate complex arithmetic have long been neglected. Here, we aimed to explore the brain mechanisms related to this difficulty effect in the case of complex addition, using event-related potential-based methods. Following previous path-finding studies, we used the inequality paradigm and different split sizes to induce the use of two approximate strategies for different difficulty levels. By comparing dependent variables from the medium- and large-split conditions, we anticipated being able to dissociate the effects of task difficulty based on approximate strategy in electrical components. In the fronto−central region, early P2 (150–250 ms) and an N400-like wave (250–700 ms) were significantly different between different difficulty levels. Differences in P2 correlated with the difficulty of separation of the approximate strategy from the early physical stimulus discrimination process, which is dominant before 200 ms, and differences in the putative N400 correlated with different difficulties of approximate strategy execution. Moreover, this difference may be linked to speech processing. In addition, differences were found in the fronto-central region, which may reflect the regulatory role of this part of the cortex in approximate strategy execution when solving complex arithmetic problems.

An approximate strategy can be defined as the process of finding an approximate answer to an arithmetic problem, without computing the exact answer^1^. Approximate strategies are important not only in daily life, but also because they are the crucial component of mathematical cognition. Previous studies have demonstrated that understanding the mechanism by which humans process approximate strategies can shed light on how more complex mathematical concepts, relationships, and strategies are acquired^2^,^3^,^4^. Although these studies have explored cognitive processing and brain function localization during execution of approximate strategies, from different perspectives, few studies have explored the effect of difficulty levels (“difficulty effect”) on the cognitive processing of an approximate strategy. Elucidating this effect would contribute to identifying an index of the efficiency of approximate strategy execution, which may be a useful neural marker for identifying groups of learners at risk of experiencing difficulty with mathematics. Therefore, we here focus on exploring the differences of evoked potentials in the brain when an approximate strategy is executed for solving arithmetic problems of different difficulty levels, using event-related potentials (ERPs). ERPs are a direct measure of the neural activity that follows in response to a specific event, with high temporal resolution, and are widely used to explore arithmetic cognition^3^,^4^.

Approximate strategy is related to exact strategy. Previous studies have addressed the dependence of cognitive processing and brain function location on the difficulty of exact strategies using several techniques commonly used in cognitive neuroscience, such as ERPs, functional magnetic resonance imaging (fMRI), and transcranial magnetic stimulation (TMS)^3^. For example, earlier ERP-based studies on brain function during addition and subtraction found that the late positive complex (LPC) is an important indicator of the difficulty effect, with greater difficulty producing more negative tendencies^5^,^6^,^7^. However, these indices cannot be regarded as an index of the difficulty of executing approximate strategies. Many studies have indicated that exact strategies differ from approximate strategies, whether in the time course of cognitive processing, or in the location of brain function. For example, using ERPs, previous studies have demonstrated that an approximate strategy may elicit more positive LPCs^8^,^9^,^10^. Dehaene *et al*. found that the regions activated by approximate arithmetic were mostly located in the visuo-spatial areas of the brain, such as bilateral parietal regions or the posterior occipital lobes, whereas brain regions activated by exact arithmetic were more consistent with language-specific areas, such as the left frontal lobes^11^. These distinctions were confirmed by other studies^12^,^13^,^14^. A study of brain-damaged patients further confirmed these conclusions; inferior parietal lobe-damaged patients tended to show poor approximate arithmetic, but had no impairment in exact arithmetic. In contrast, left frontal lobe-damaged patients showed poor exact arithmetic abilities, but demonstrated no impairment in executing approximate arithmetic^14^,^15^. These studies prove that approximate strategy and exact strategy processing use distinct brain pathways. Moreover, Liu recently demonstrated that computationally, cognitively, and structurally distinct processes are used in the execution of exact strategies versus approximate strategies^16^. Given these findings, we believe that the factors related to approximate strategy execution involving various difficulty levels of arithmetic problems should now be investigated.

Previous studies have attempted to investigate this problem using simple arithmetic. For example, Stanescu *et al*. used simple arithmetic as problem material, and problem size as the variable determining difficulty, and identified differences in cognitive processing and location, depending on whether participants used exact or approximate strategies^17^. ERP results have revealed a change that correlates with difficulty appearing by 168 ms. This signal was mainly located in the left anterior, inferior frontal areas, which are used for executing exact strategy. With a change in difficulty level, the signal appeared after 384 ms and was mainly located in the fronto-central area, indicating the use of approximate arithmetic strategies. By recording blood oxygenation level dependent (BOLD) signals, they found that the left-lateralized signals (from the left intraparietal, left precentral, and left inferior frontal regions) are modulated by the level of difficulty for exact strategies, but there were no locations specifically sensitive to difficulty levels when implementing approximate strategies. To our knowledge, except for the study cited above, no other studies have explored the brain mechanisms responding to differences in difficulty levels during approximate strategy execution. However, among studies using simple arithmetic problems, many have demonstrated that participants usually obtain the answer directly by fact retrieval from memory, and that cognitive processing is automatic^18^,^19^. In other words, irrespective of the paradigm adopted by researchers to induce participants to employ exact or approximate strategies for solving simple arithmetic problems, the fact retrieval system may confound the results obtained^20^,^21^. In addition, previous studies found differences in the brain mechanisms involved in solving simple and complex arithmetic problems; for example, the left hemisphere is dominant in simple arithmetic processing, but complex arithmetic processing requires a synergy between left and right hemispheres^22^,^23^. Therefore, the present study used complex arithmetic (addition) as task material for exploring brain mechanisms underlying computing of approximate solutions to problems of controlled but variable difficulty.

A body of literature has demonstrated that people select and execute different strategies based on the characteristics of the problems during arithmetic cognition^24^. The present study uses the findings of previous studies^9^,^10^ for inducing different levels of difficulty, while retaining an approximate strategy. El Yagoubi *et al*. used a large−small split to induce a separation between exact strategy and approximate strategy^9^,^10^; this effect was confirmed by many other studies^8^,^25^. Specifically, the required arithmetic correct sums were ±2% or ±5% away from 100 (e.g. 100 ± 2; 100 ± 5) as the small-split condition, and ±10% or ±15% away from 100 (e.g. 100 ± 10; 100 ± 15) as the large-split condition. Under these conditions, participants tended to adopt exact arithmetic strategies to solve small-split problems and approximate arithmetic strategies to solve large-split problems. Behavioral evidence, such as task performance characteristics, including speed−accuracy tradeoff, shows that low speed and a high error rate characterize exact arithmetic processing, whereas high speed and a low error rate characterize approximate arithmetic processing. This can be used to infer the strategy used by participants. Combining ERP evidence with performance/strategy data suggests the separation of strategies when the second number was presented within 250 ms of presentation of the first number, and that the differences observed in the range of 250–600 ms reflect cognitive differences between the execution processes of the two strategies. Based on this concept, and since 100 ± 10 or ±15 split solvers tended to adopt approximate strategies, we believe that 100 ± 20 or ±25 split solvers will adopt approximate strategies a fortiori. Therefore, inclusion of two splits, 100 ± 10 or ±15, and 100 ± 20 or ±25, will allow this study to access two difficulty levels without inducing a confounding change of strategy. However, the premise of this hypothesis is that Chinese subjects will behave similarly to those in the study by El Yagoubi *et al*. Previous studies have found cross-cultural inconsistencies in arithmetic cognitive strategies^9^,^10^. Therefore, this study employed three conditions, namely a small split (100 ± 2 or ±5), medium split (100 ± 11 or ±15), and large split (100 ± 21 or ±25). The contrast between the small and medium split can be used to verify the conclusion of El Yagoubi *et al*. for the case of Chinese participants^9^,^10^, while the contrast between the medium and large split can be used to explore difficulty effects in approximate problem solving.

## Results

### Behavioral results

ANOVA of reaction time (RT) data showed a significant main effect among small (848.663 ± 63.386 ms; which refers to mean and standard deviations), medium (652.327 ± 44.922 ms), and large (615.494 ± 37.456 ms) splits (*F*_(2,30)_ = 36.589, *P* < 0.001). Multiple comparison analysis of the three conditions showed significant differences between the reaction times for small-split and medium-split (mean difference = 196.337 ± 31.420 ms, *P* < 0.001), and between medium-split and large-split (mean difference = 36.832 ± 14.779 ms, *P* = 0.037).

Repeated-measures ANOVA of the accuracy rate found a significant main effect of reaction time among small- (82.60 ± 2.28%), medium- (97.43 ± 0.60%) and large- (96.93 ± 1.25%) split tasks (*F*_(2,30)_ = 36.589, *P* < 0.001). Further multiple comparisons found a significant difference between the accuracy rate of small-split and medium-split tasks (mean difference = −13.00 ± 2.70%, *P* < 0.001), but not between medium-split and large-split tasks (mean difference = −0. 50 ± 1.11%, *P* = 0.665).

### Differences in brain mechanism between exact and approximate arithmetic

ANOVA for midline electrodes (Table 1) revealed that the interaction of split and electrode site was significant only in the 1400–1700 ms epoch (*F*_(3, 36)_ = 4.156, *P* = 0.027, *η*^*2*^ = 0.257). When we analyzed the main effect, we found that a small-split effect (−3.793 ± 0.869 μV) induced a significantly greater negative wave than did a medium-split effect (−2.293 ± 0.841 μV), at the Cz electrode (*F*_(1,15)_ = 14.816, *P* = 0.002, *η*^*2*^ = 0.553).

ANOVA for lateral electrodes (Table 1) found that the interaction of split and hemisphere was significant at 1050–1400 ms (*F*_(2,30)_ = 7.707, *P* = 0.008, *η*^*2*^ = 0.391). In more detail, simple main effect analysis showed that the medium-split effect (0.553 ± 0.213 μV) induced a significantly greater negative wave than did the small-split effect (0.376 ± 0.183 μV), but only in the right hemisphere (*F*_(1,15)_ = 5.578, *P* = 0.036, *η*^*2*^ = 0.317). In addition, the interaction of split effect and brain region (frontal, central, parietal) was also significant in this time window (*F*_(2,30)_ = 7.707, *P* = 0.008, *η*^*2*^ = 0.391). Further simple effect analysis of the results showed that the small-split effect induced a greater negative wave than did the medium-split effect in the frontal (*F*_(1,15)_ = 4.785, *P* = 0.049, *η*^*2*^ = 0.285) and parietal regions (*F*_(1,15)_ = 15.734,*P* = 0.002, *η*^*2*^ = 0.567) (frontal region: small, 0.551 ± 0.794 μV; medium, 0.106 ± 0.715 μV; parietal region: small, 0.040 ± 0.537 μV; medium, 0.571 ± 0.487 μV). The interaction of the split effect and brain region (frontal, central, parietal) was significant in the 1400–1700 ms epoch (*F*_(2,30)_ = 5.339, *P* = 0.020, *η*^*2*^= 0.308). Further analysis found a significant simple main effect only in the parietal lobe (*F*_(1,15)_ = 8.037, *P* = 0.015, *η*^*2*^ = 0.401), and that a small-split effect (−0.302 ± 0.479 μV) induced a greater negative wave than did a medium-split effect (0.270 ± 0.474 μV). The interaction of the split and brain region (frontal, central, parietal) was significant in the 1700–2000 ms epoch (*F*_(2,30)_ = 3.957, *P* = 0.044, *η*^*2*^ = 0.248). Further analysis revealed a significant simple main effect only in the parietal lobe (*F*_(1,15)_ = 4.728, *P* = 0.050, *η*^*2*^ = 0.283), and that a small-split effect (−0.518 ± 0.453 μV) induced a greater negative wave than did a medium-split effect (0.025 ± 0.364 μV).

### Brain mechanism of approximate arithmetic difficulty effects

To explore the difference of the mean amplitude in each ROI for approximate arithmetic difficulty effects, we first performed a global repeated-measures ANOVA with factors Time (6 levels), Ant-Pos (4 levels), Lateral (3 levels), and Split (2 levels). The results showed three interactions with the split factor (Split × Lateral, F(2,30) = 3.900, P = 0.034, *η*^*2*^ = 0.275; Split × Ant-Pos × Time, F(15,225) = 6.287, P = 0.020, *η*^*2*^ = 0.309; Split × Ant-Pos × Lateral × Time, F(30,450) = 5.931, P = 0.014, *η*^*2*^ = 0.290). Given the significant interaction of Split × Ant-Pos × Lateral × Time, it can be speculated that the difference between medium and large splits may appear in different ROIs and time windows. Based on the above analyses, we performed two-way repeated-measures ANOVAs (time × split) in each of the ROIs.

It can be seen from Table 2 that the split main effect, and the split effect, by the time window interaction on the average amplitude was significant at the middle fronto-central position. Furthermore, a significant interaction of the split effect and the time window was also clear at the right centro-parietal site. The results of the simple main effect are shown in more detail in Table 3.

The simple main effect at the middle fronto-central site was significant in epochs 950–1050 ms, 1050–1200 ms, and 1200–1500 ms, and the medium split induced a significantly greater negative wave than did the large split in all three time-windows.

## Discussion

The behavior results showed that with an increase in the split, the mean RT showed a declining trend, and the split contrasts (small vs. medium and medium vs. large) were both significant. These results demonstrated that the three splits formed a clear difficulty gradient. It is worth noting that the difficulty gradient is not linear, i.e., the medium/small-split difference (196 ms) was significantly larger than the large/medium-split difference (36 ms). However, in terms of the split size, the difference between the small and medium splits tended to be equal to the differences between the large and medium split problems. This result may reflect the fact that cognitive processing of small-split effects is much more complex than that of medium- or large-split effects. The analysis of accuracy rates indicated that accuracy tended to improve significantly with the increase in the split effect. However, significant differences only appeared between small and medium splits, and accuracy rates were almost equal between medium and large splits. When we combined the accuracy data with reaction time, we found that, in terms of small and medium splits, the reaction time decreased significantly and accuracy rates increased significantly as the split effect increased, and there was a clear speed−accuracy imbalance. In terms of medium and large splits, although reaction time appeared to decline significantly with an increase of the split effect, it decreased significantly less, with no difference in accuracy, leading to no observed speed-accuracy imbalance. The speed−accuracy imbalance in small and medium splits was consistent with previous research results^8^,^9^,^10^, which may reflect that the small- and medium-split effects drove adoption of completely different strategies^9^,^10^. In other words, Chinese subjects also tend to use an exact strategy for solving small-split problems and an approximate strategy for solving medium-split problems. In terms of the medium- and large-split contrast, the accuracy rates were virtually the same, although the difference in reaction time was highly significant. This may reflect that participants tended to use the same strategy—the approximate strategy—for solving medium- and large-split problems, with large split being easier than medium split problems (i.e., different levels of difficulty).

We have discussed the separation of exact and approximate strategies, based on small- vs. medium-split problems. The speed−accuracy imbalance seen in the behavioral results for the small- and medium-split contrast were in agreement with previous research, possibly reflecting the separation of the two strategies^9^,^10^. Further analysis of the ERP results showed no difference in the averaged amplitudes between small and medium splits in the 0–300 ms, 300–800 ms, and 800–1050 ms epochs. The results for 0–300 ms (“+” presented) and 300–800 ms (second operand presented) were consistent with those of El Yagoubi *et al*., which suggested that subjects did not preselect strategies before the second operand was presented^9^,^10^. The second operand started to be presented at the 800 ms epoch, and the first time window of 0–250 ms that followed presentation of the second operand also belonged to the physical stimulus-identification stage; there was no significant difference in the physical stimulus stage. From 1050 to 1400 ms (250–600 ms after the second operand was presented), difference between the small and medium split began reaching significance in the right hemisphere. This difference may be caused by a quantitative representation imbalance between the left and right hemispheres in a comparison task^26^,^27^,^28^. In addition, there was a difference between small- and medium-split effects in the frontal and parietal lobes in the 1050–1400-ms epoch, and the difference seen at the frontal sites may be caused by the demands of the small/medium split on working memory. Because small splits drove adoption of an exact arithmetic strategy, it increased memory load. However, the medium split drove adoption of an approximate arithmetic strategy, with a relatively low working memory burden. Therefore, the small/medium-split contrast would be likely to correlate with an activation difference in the frontal lobe, while the difference in the parietal lobe could be explained by previous studies. It was previously reported that the parietal area is the key location that reflects strategy execution between exact and approximate strategies in arithmetic processes^5^,^6^,^7^,^29^,^30^. Similarly, separation of small- and medium-split brain mechanisms in the parietal lobe in the 1400–1700 ms and 1700–2000 ms epochs could both be interpreted as reflecting exact versus approximate arithmetic strategies.

These results proved that, even after changes in task details, small and medium splits were associated with exact and approximate arithmetic strategies, respectively, consistent with the reports by El Yagoubi *et al*.^9^,^10^. These results confirmed that Chinese subjects use exact and approximate strategies, respectively, in solving small- and medium-split problems. Based on the above conclusion, combined with the behavioral data, we deduced that subjects use the approximate strategy for solving large-split problems. Therefore, it is possible to study the brain mechanisms involved in solving problems of different difficulty levels using approximate arithmetic strategies, by investigating the difference between medium- and large-split effects.

Combining the behavioral results for the medium/large-split contrast, we further analyzed the average amplitude differences under two conditions to investigate the brain-mechanistic differences related to the difficulty effect in approximate arithmetic problem solving. Overall analysis showed that medium-split problems elicited a significantly more negative wave than large-split problems in middle fronto-central sites. This was also supported by the results of a previous study. Stanescu *et al*. used simple arithmetic to show the sensitivity of ERPs in fronto-central sites for assessing different levels of difficulty in executing approximate strategies^17^. Furthermore, a comparison of children with developmental dyscalculia and normal controls demonstrated that the middle frontal gyrus plays a key role in approximate arithmetic^13^. These studies seemed to show that the fronto-central cortex participates in the response to different difficulty levels during the execution of approximate strategies in complex arithmetic. What is the regulating mechanism in this cortex? Previous reports have implicated the middle frontal area, over mainly the dorsal or middle frontal gyrus in the frontal lobe, as related to working memory^31^,^32^,^33^,^34^. Some previous studies, such as that by Kalaman and LeFevre^12^, have indicated that exact arithmetic is more dependent on working memory than is approximate arithmetic. The present study suggests that, in addition, working memory also has a regulating effect in the response to the difficulty level during approximate complex arithmetic solving.

**Figure 1:**
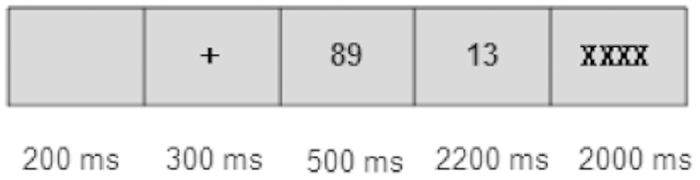
Timing diagram of stimulus sequence; “89” and “13” are examples.

The analysis of each time window revealed that the 800–1050 ms epoch (0–250 ms after second operand onset) contained an obvious N1-P2 complex, the N1 time window was “800–950 ms” (1–150 ms after second operand onset), and the P2 time window was “950–1050 ms” (150–250 ms after second operand onset). In previous studies, N1-P2 waves have been regarded as early components of the mental arithmetic process and have been interpreted in two main ways. Some researchers have interpreted the N1-P2 waves observed during mental arithmetic tasks as reflecting the level of difficulty of different tasks in psychological processing^29^. However, Näätänen^35^ believed that the early N1-P2 composite wave reflects only the visual encoding process^36^,^37^,^38^. The question remains as to which interpretation is reasonable. Comparison of our ERP data with the experimental design (Fig. 1) shows that presentation of “+” (0–300 ms), the first operand (300–800 ms), and the second operand all elicited N1-P2 components in the central and parietal lobes, which is consistent with the results of Näätänen^35^. This indicates that the early N1-P2 components may reflect visual encoding of operands and objects. However, in this study, we found a difference in the ERP response between medium- and large-split problems in the time window of 950–1050 ms (i.e., P2), which may indicate that the N1-P2 waves, especially the P2 wave, reflect the level of difficulty of different tasks during psychological processing, which is supported by the study by Iguchi & Hashimoto^29^. Accordingly, we may conclude that N1-P2 waves not only reflect the process of visual encoding of objects, but also the different difficulty levels of executing the approximate strategy. Kong *et al*. found that the P2 wave could be differentially induced by problems of different sizes^39^, supporting the conclusion of this study that the difference in P2 that was present in the fronto-central region may be the important early index of approximate arithmetic difficulty. However, it also reflects the role of the central fronto-central area in regulating difficulty effects in approximate arithmetic problem solving.

The negative wave seen in the 1050–1500 ms epoch (250–700 ms after the second operand onset) has often been regarded as part of the slow wave in the study of mental arithmetic, but it has also been assigned to the N400 complex, which has been implicated in language processing^36^,^37^,^38^. If the epoch were considered as part of the slow wave in this study, it could be considered as reflecting different difficulty levels of arithmetic processing tasks. Previous studies have indicated that a slow wave elicited in this time window reflects the brain mechanism involved in exact arithmetic difficulty, especially if located in the parietal lobe^5^,^29^,^30^. This study also found this difference in the same time window. Accordingly, we could conclude that, as with previous studies on difficulty levels in exact arithmetic processing, the slow wave is an important index that reflects the differential difficulty of execution among approximate strategies in arithmetic processing. However, it is usually located in the middle fronto−central region, rather than in the parietal lobe. In addition, from the perspective of semantic processing, previous research on simple exact and approximate arithmetic has shown that the exact strategy relies mainly on the language-representation and related regions, but the approximate strategy is not related strongly to this area^11^. Pesenti *et al*. confirmed that simple exact arithmetic was related to the verbal representation region and its closely allied regions, such as the inferior frontal gyrus, the superior temporal gyrus, and so on^40^. Research in aboriginal participants has indicated that this group, using the exact strategy, could only solve arithmetic problems in which the number can be represented in words, but that they are not limited by words when solving arithmetic using approximate strategies^41^,^42^. All of these studies have indicated that execution of the approximate strategy is less reliant on the brain’s language region than on execution of the exact strategy. However, the evidence for this conclusion has been entirely based on simple arithmetic to date. Zago *et al*. have indicated that it is difficult to separate the true exact strategy and the real operation using simple arithmetic tasks^43^, suggesting that the true exact strategy is closely related to the semantic processing network. This emphasizes the need for caution when concluding that approximate arithmetic does not rely on the semantic processing system, when based on studies that used only simple arithmetic tasks. In the present study, if the negative slow wave at 250–700 ms is interpreted as N400, it seems to suggest that different difficulty levels in executing the approximate strategy are closely related to language cognitive processing when solving complex arithmetic problems. We also need to remain cautious because the waveform observed around the time of N400 was not typical of N400. To confirm the relation between approximate strategy execution and language cognitive processing, further studies are required.

Based on the above analysis, some conclusions can be drawn. Firstly, in the early stage of mental arithmetic, the dissociation of difficulty of approximate strategy execution starts around 200 ms, and P2 is an important landmark. Secondly, the ERP slow component in the 250–700 ms epoch may reflect the difficulty of processing the approximate strategy. If this epoch is understood as N400, which is implicated in semantic processing, we may conclude that there is a close connection between approximate arithmetic difficulty and verbal processing. Thirdly, the early component, P2, and the late slow component both appeared in the middle fronto−central region, implicating this cortical region in regulating the difficulty effect when executing approximate strategies for solving arithmetic problems.

## Methods

### Participants

Sixteen undergraduate students (nine males and seven females; mean age 20 y; range: 18–23) were recruited from freshman, sophomore, and junior years at the South China Normal University. All subjects were right-handed, and had normal or corrected-to-normal eyesight and had no history of neurological or psychiatric disorders. Considering that participant arithmetic ability could affect our experimental results, we limited participants to students majoring in mathematics or related disciplines. An additional inclusion criterion was that the mathematics score was confined to a range of 90 to 110 from a total of 150 on the college entrance examination. All participants were paid for their participation. All participants provided written informed consent before the start of the experiments. This study was approved by the Research Ethics Committee of the South China Normal University and the methods were carried out in accordance with the approved guidelines. Informed consent was obtained from all participants.

### Stimuli

The stimuli were 180 addition arithmetic problems, presented in a standard form (i.e., a + b), with the operands “a” and “b” being two-digit numbers. Numbers were displayed at the center of a computer screen, and participants were asked to decide whether the result was smaller than 100. All arithmetic results were in one of the categories large-split, medium-split, or small-split. For small-split problems, the correct answers were ±2 (e.g., 89 + 13, 87 + 12) or ±5 (e.g., 89 + 16, 83 + 12) away from 100. For medium-split problems, the correct answer were ±11 (e.g., 73 + 16, 93 + 18) or ±15 (e.g., 62 + 23, 81 + 34) away from 100. For large-split problems, the correct answers were ±21 (e.g., 53 + 26, 32 + 89) or ±25 (e.g., 56 + 19, 97 + 28) away from 100.

We chose 60 questions from large-, medium- and small-split categories, without repeating any problem. Based on previous research, problems were selected according to several constraints, in order to avoid a number of potential confounds^15^,^44^,^45^. First, answers were balanced between problems yielding answers greater than or less than 100. Second, the side of the summation expression with the larger operand was controlled by having the first operands being the larger in half of the problems. Third, no operand had a units-digit equal to 0 or 5 (e.g., 60 + 38 or 65 + 33). Finally, no problems had operands repeating the same units digit (e.g., 78 + 18).

### Procedure

Participants were seated comfortably at 60 cm away from the front of a computer screen, at eye level, directly facing the screen center. Participants were instructed to solve the problems mentally, as quickly and accurately as possible, as soon as the second number was presented. They were asked to press the “F” button if the sum was larger than 100, and the “J” button if the sum was smaller than 100. Response hands were counterbalanced across participants. The set of 180 problems was divided into four blocks of 45 problems each, and these problems were randomized for each participant within each block and between blocks.

The experimental procedure was controlled by E-Prime 2.0 software, and a schematic diagram of the procedure is shown in Fig. 1. A warning-fixation stimulus was displayed in the center of the screen for 200 ms, then the screen showed a “+” for 300 ms, followed by the first operand, displayed for 500 ms. Then the second operand replaced the first operand, and participants were given 2200 ms from second operand onset to provide their answer. If participants provided the answer within 2200 ms, the second operand disappeared, being replaced by four Xs. If the answer was not provided within 2200 ms, the program showed the four Xs and recorded a wrong answer. Participants were asked to refrain from blinking, shaking, or moving during the critical phase of EEG recording. The four Xs informed participants that they could blink and move their eyes. Each block took about 3 min and participants were required to rest 1–2 min after each block to relieve fatigue, resting twice during the experiment.

### Data acquisition and analysis

We recorded and analyzed ERPs using an EEG system made by Germany Brain Products (Brain Products, Munich, Germany). Participants wore a cap fitted with 64 electrodes arranged according to the 10–20 international system, referenced online to the left mastoid and rereferenced to the average of the left and right mastoids offline. The vertical electrooculograms (EOG) were recorded supra- and infra-orbitally at the left eye. The horizontal EOG was recorded from the left versus right orbital rim. The scalp impedance and the impedance between the electrodes was less than 5 kΩ. The signal was filtered at 0.01–30 Hz and epoched to cover the range of −100 to 3200 ms. ERP correction was performed with a −100 to 0 ms baseline before stimulus onset. An automatic ocular correction procedure was used to eliminated EOG artifacts by the Vision Analyzer software with one sensor as the EOG monitor and the other as the reference for both the horizontal and vertical EOG sensor pairs. Trials with a mean EOG voltage that exceeded ±80 μV were regarded as being contaminated by artifacts, and were excluded from averaging. In addition, the signal was recorded at a sampling frequency of 500 Hz and was processed offline using Brain Electromagnetic Source Analysis software.

### Controlling arithmetic strategy (exact versus approximate)—analysis of small- and medium-split effects on ERPs

**Figure 2:**
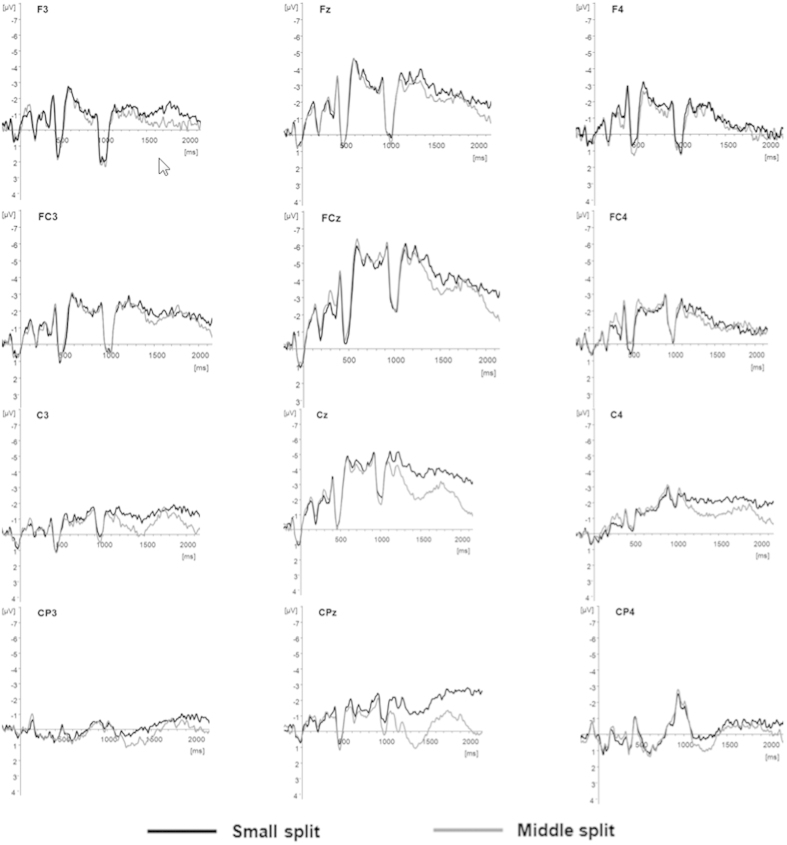
Grand averaged event-related potentials (ERPs) elicited at the midline, lateral electrodes during small split and middle split arithmetic.

To verify the previous reports on strategy control, we analyzed the effect of the small- versus medium-split contrast on ERPs. The time windows and brain regions were consistent with previous studies^9^,^10^. Setting the ERP total average amplitude as the dependent variable, we compared the average amplitude in response to the small-split task with that in response to the medium-split task in the midline electrodes (Fz, Cz, Pz, and Oz, a total of four electrode locations) (Fig. 2) and in the lateral electrodes (AF4, AF3, F3, F7, F4, F8, C3, FC5, T7, C4, FC6, T8, CP1, CP5, P3, CP2, CP6, and P4). The midline electrodes were analyzed using a split effect (small, medium) × electrode location (Fz, Cz, Pz, and Oz) in a two-factor repeated-measures analysis of variance (ANOVA). The lateral electrodes were analyzed using split effect (small, medium) × brain hemisphere (left, right) × regions (frontal: F3, F7, FC1, F4, F8, and FC2; central: C3, FC5, T3, C4, FC6, and T4; and parietal: CP1, CP5, P3, CP2, CP6, and P4) in a three-factor repeated-measures ANOVA. The time window of analysis was 0–2000 ms. All results used Greenhouse−Geisser parameters to revise the *P* values, while the Bonferroni−Holm method was used to correct for multiple testing.

### Correlates of approximate strategy difficulty analysis of medium-and large-split effects on ERPs

**Figure 3:**
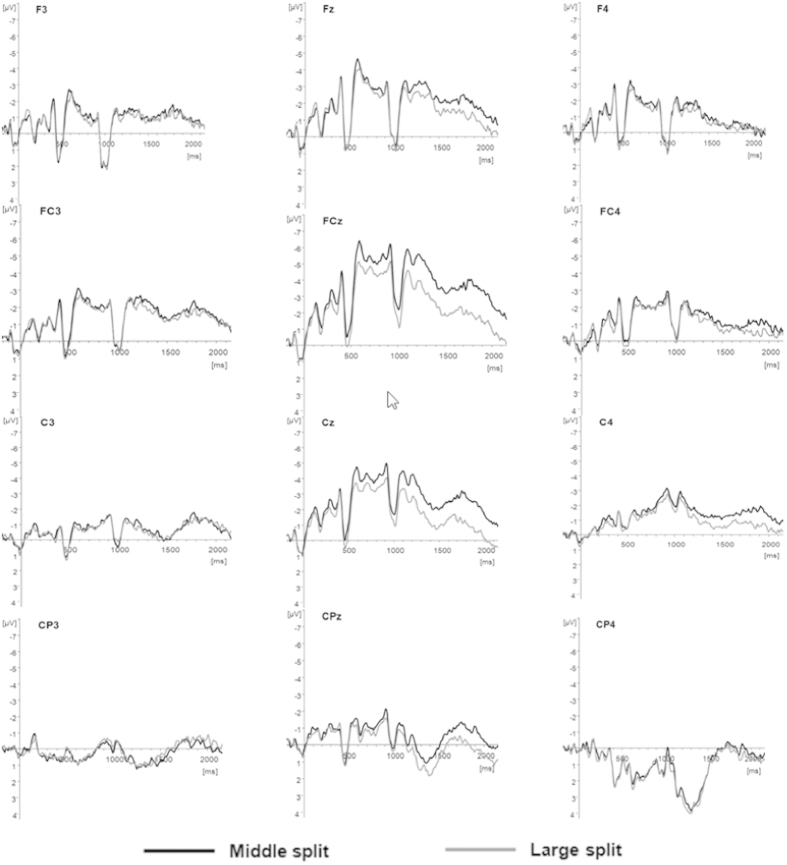
Grand averaged event-related potentials (ERPs) elicited at the midline, lateral electrodes during small split and medium split arithmetic.

According to previous studies and combing the waveform in the present study, we chose the 2000-ms time window as the data analysis comprised the following epochs: “plus-sign” rendering time (0–300 ms), first-number rendering time (300–800 ms),150 ms after the first number presented (800–950 ms), 150–250 ms after the second number presented (950–1050 ms), 250–400 ms after the second number presented (1050–1200 ms), 400–700 ms after the second number presented (1200–1500 ms), 700–900 ms after the second number presented (1500–1700 ms), and 900–1200 ms after the second number presented (1700–2000 ms). To avoid failure of statistical validity, this study used repeated-measures analysis of variance to analyze data across multiple channels and multiple time windows. Following previous studies^46^,^47^, the entire set of scalp electrode sites was locally pooled to form 12 topographical regions of interest (ROIs)(Fig. 3). The classification rules were: anterior−posterior was divided into four levels, and left−right was divided into three levels, to form a total of 12 ROIs. These were: left frontal (AF7, F7, F3), middle frontal (FPz, AF3, AF4, F1, Fz, F2), right frontal (AF8, F8, F4), left fronto−central (FT7, FC5, T7, C5), middle fronto−central (FC1, FCz, FC2, C1, Cz, C2), right fronto−central (FT8, FC6, T8, C6), left centro−parietal (TP7, CP5, P7, P5), middle centro−parietal (CP1, CPz, CP2, P1, Pz, P2), right centro−parietal (TP8, CP6, P8, P6), left parieto−occipital (PO7, PO3, O1), middle parieto−occipital (Oz, POz), and right parieto−occipital (PO8, PO4, O2).

To explore the difference between medium- and large-split problems in ERPs, we performed two-step analyses as in previous studies^46^,^48^,^49^. Firstly, we calculated the mean voltages within the ROIs, which were used as dependent variables in repeated-measures ANOVAs. The independent variables included Time−Window (Time), Anterior−Posterior (Ant-Pos, 4 levels), Left−Right (Lateral, 3 levels), and Split problems (Split, 2 levels). A global ANOVA with four independent variables (Time, Ant-Pos, Lateral, Split) and one dependent variable (mean voltage) was performed. Secondly, according to the interactions with the split, we executed ANOVAS for different ROIs, separately. All results used Greenhouse−Geisser parameters to revise the *P* values and the Bonferroni−Holm method was used to correct for multiple testing.

## Additional Information

**How to cite this article**: Xiang, Y.h. *et al*. Brain-mechanistic responses to varying difficulty levels of approximate solutions to arithmetic problems. *Sci. Rep*. **6**, 24194; doi: 10.1038/srep24194 (2016).

## Figures and Tables

**Table 1:** Variance analysis of brain average amplitude during small and medium split effects (n = 16).

Time window	Midline			Lateral						
	A	B	AB	A	C	AC	D	AD	CD	ACD
0–300 ms	−	+	−	−	−	−	+	−	−	−
300–800 ms	−	+	−	−	−	−	+	−	−	−
800–1050 ms	−	+	−	−	+	−	+	−	−	−
1050–1400 ms	−	+	−	−	+	+	−	+	−	−
1400–1700 ms	−	+	+	−	−	−	−	+	−	−
1700–2000 ms	−	+	−	−	−	−	−	+	−	−

Note: (+) significant effect (*P* < 0.05); A, split; B, electrode; C, hemisphere; D, localization; 0–300 ms, warning fixation stimulus; 300–800 ms, first operand display; second operand shown 800 ms after warning signal.

**Table 2:** ANOVA of split effect and time window in various brain areas: average amplitude (n = 16).

Location	Split effect			Split effect × time window		
	*F*	*P*	*η*^*2*^	*F*	*P*	*η*^*2*^
Left frontal	0.001	0.984	0.000	0.444	0.699	0.036
Middle frontal	0.120	0.735	0.010	0.247	0.946	0.020
Right frontal	0.033	0.858	0.003	0.745	0.542	0.058
Left fronto-central	0.875	0.368	0.068	0.512	0.633	0.041
Middle fronto-central	4.644	0.030*	0.279	3.038	0.016*	0.202
Right fronto –central	0.780	0.394	0.061	0.667	0.522	0.053
Left centro-parietal	1.196	0.296	0.091	1.137	0.307	0.087
Middle centro-parietal	0.344	0.569	0.028	0.451	0.811	0.036
Right centro-parietal	1.032	0.330	0.097	2.355	0.050*	0.153
Left parieto-occipital	0.560	0.467	0.041	0.248	0.766	0.019
Middle parieto-occipital	0.350	0.564	0.026	0.358	0.724	0.027
Right parieto-occipital	0.010	0.920	0.001	0.863	0.477	0.062

Note: *F, F* value; *P*, significance level; *η*^2^, partial *η*^2^.

^*^P < 0.05, **P < 0.01.

**Table 3:** Simple effect analysis of split effect and time window (n = 16).

Time window	Middle fronto-central			Right centro-parietal		
	*F*	*P*	*η*^*2*^	*F*	*P*	*η*^*2*^
0–300 ms	1.231	0.289	0.093	0.996	0.338	0.077
300–800 ms	3.720	0.078	0.237	0.362	0.561	0.035
800–950 ms	4.165	0.064	0.258	1.546	0.237	0.114
950–1050 ms	6.826	0.023*	0.363	0.002	0.969	0.000
1050–1200 ms	4.858	0.046*	0.272	0.749	0.404	0.059
1200–1500 ms	5.273	0.040*	0.305	0.887	0.365	0.069
1500–1700 ms	1.231	0.289	0.093	0.836	0.379	0.065
1700–2000 ms	2.320	0.098	0.157	2.057	0.177	0.146

Note: *F*, *F* value; *P*, significance level; *η*^2^, partial *η*^2^.

^*^P < 0.05, **P < 0.01.
