# Erratum: Brain-mechanistic responses to varying difficulty levels of approximate solutions to arithmetic problems

**DOI:** 10.1038/srep33620

**Published:** 2016-10-03

**Authors:** Yanhui Xiang, Yiqi Jiang, Xiaomei Chao, Qihan Wu, Lei Mo

Scientific Reports 6: Article number:2419410.1038/srep24194; published online: 04 13 2016; updated: 10 03 2016

In this Article, the legend of Figure 3 is incorrect:

Grand averaged event-related potentials (ERPs) elicited at the midline, lateral electrodes during small split and medium split arithmetic.

Should read:

Grand averaged event-related potentials (ERPs) elicited at the midline, lateral electrodes during middle split and large split arithmetic.

